# Protective effect of Zhuyeqing liquor, a Chinese traditional health liquor, on acute alcohol-induced liver injury in mice

**DOI:** 10.1186/1476-9255-10-30

**Published:** 2013-10-03

**Authors:** Hong-ying Gao, Jian Huang, Hang-yu Wang, Xiao-wei Du, Suo-ming Cheng, Ying Han, Li-fei Wang, Guo-yu Li, Jin-hui Wang

**Affiliations:** 1 School of Traditional Chinese Materia Medica 49#, Shenyang Pharmaceutical University, Wenhua Road 103, 110016 Shenyang, P. R. China; 2 School of Pharmacy, Shihezi University, 832002 Shihezi, P. R. China; 3 Shanxi Xinghuacun Fen Jiu Group Co., Ltd, 450000 Shanxi, P. R. China

**Keywords:** Zhuyeqing liquor, Hepatoprotective effect, Alcohol, Acute liver injury

## Abstract

The study first evaluated the hepatoprotective effect of Zhuyeqing Liquor (ZYQL) against acute alcohol-induced liver injury in mice. Animals were administered orally with 50% alcohol 12 ml/kg at 4 h after the doses of ZYQL everyday for fourteen consecutive days except mice in normal group. The protective effect was evaluated by biochemical parameters including serum aspartate transaminase (AST), alanine transferase (ALT), total-bilirubin (TBIL) and reduced glutathione (GSH), malondialdehyde (MDA), superoxide dismutase (SOD) in liver tissue. The result were confirmed histopathologically and the expression of TNF-α in mice liver was determined by immunohistochemistry analysis. HPLC-PDA was used for phytochemical analysis of ZYQL, and the plant source of each compound was claritied by UPLC-TOF-MS. The result showed that pretreatment with ZYQL exhibited a significant protective effect by reversing the biochemical parameters and histopathological changes in a dose depended manner. HPLC analysis indicated that ZYQL contained flavonoids, iridoids, terpenoids and phenolic acids, which might be the active chemicals. This study demonstrated the hepatoprotective activity of ZYQL, thus scientifically supported the function of its health care.

## Introduction

Alcohol is a dietary component which is usually consumed for its psychophysical and mood-altering effects. However, long-term alcohol consumption may cause damage to vital organs including gastrointestinal, cardiovascular, endocrine and central nervous systems [1,2], especially the liver disease which has become a social problem. In China, the incidence of alcoholic liver disease (ALD) has increasing and becoming an important risk factor for morbidity and mortality in addition to viral hepatitis [3], due to the increased frequency of drinking and change of diet construction. Drugs including bifendate, tiopronin and bicyclol have been reported to have protective effect against ALD, however, definite treatment strategies for ALD remain undefined. Hence, prevention of ALD is a therapeutic challenge and developing inexpensive natural agents which possesses the hepatoprotective effects can achieve the goal is further a higher challenge, and also has become the focus of research in recent years.

Zhuyeqing Liquor (ZYQL) is a famous traditional Chinese functional liquor comprising twelve crude drugs: *Lophatherum gracile* Brongn. (Zhuye), *Gardenia jasminoides* Ellis (Zhizi), *Lysimachia capillipes* Hemsl. (Paicao), *Angelica sinensis* (Oliv.) Diels (Danggui), *Kaempferia galanga* L. (Shannai), *Citrus reticulata* Blanco (Chenpi), *Chrysanthemum morifolium* Ramat. (Juhua), *Amomum villosum* Lour. (Sharen), *Santalum album* L. (Tanxiang), *Eugenia caryophyllata* Thunb. (Dingxiang), *Aucklandia lappa* Decne. (Muxiang), *Lysimachia foenum-graecum* Hance (Linglingxiang). This formula could trace back to the Warring States Period and it became popular among people in the South and North Dynasty. With the rapid social development, it had reached its climax in Tang Dyansty and Song Dynasty, and has been formally authorized as a functional health liquor in 1998 by Ministry of Public Health in China. As a functional health liquor, it has various biological properties like anti-oxidant, anti-fatigue and immunoenhancement [4], and some reports also showed that most of the medical herbs it contains have the effect of hepatic injury protection [5], blood coagulation, anti-tumor, anti-inflammation, gastrointestinal protection, immunity regulation, anti-oxygen *etc*. [6-9] and some which have been used in TCM formulation on clinical practice for their hepatoprotective effects. Our laboratory has identified the main components in ZYQL through systematically chemical isolation and detected by HPLC-PDA detectors in previous study. These main components of ZYQL was also claritied its plant source by UPLC-TOF-MS. However, there is no pharmacological study for the hepatoprotective effect of ZYQL yet, scientific literature data supporting the health function of ZYQL in ALD are unavailable and its tentative mechanisms are still unknown. Herein, evaluating the hepatoprotective effect and identifying the active group of ZYQL are important in revealing the therapeutic material basis, providing the evidence for the pharmacological effect and improving the quality control of this health liquor.

In view of that, the present study was conducted to investigate the potential protective effects of feeding ZYQL on alcohol-induced liver injury in mice. The study also aimed to explore the underlying mechanisms of such effects.

## Materials and methods

### Materials and reagents

Methanol and formic acid (HPLC grade) was purchased from Fisher Scientific Co. (Franklin, USA). Phosphoric acid (analytical grade) was purchased from Tianjin Guangfu Chemical Reagent Co. Ltd. (Tianjin, China). Water was prepared using a redistilled water equipment. Ethanol and other chemicals used were all of analytical grade and from Nanjing Chemical Co.(Nanjing China).

Alanine aminotransferase (ALT), aspartate aminotransferase (AST), total bilirubin (TBIL), glutathione (GSH), superoxide dismutase (SOD) and malondialdehyde (MDA) kits were obtained from Nanjing Jiancheng Bioengineering Institute (China). Protein assay kit was from Zhongshan Institute of Biotechnology (Beijing, China). Antibodies against TNF-α was purchased from abcam Biotechnology (abcam, UK), All other chemicals used in these experiments were of analytical grade and were obtained from commercial sources (Beijing, China).

The reference standard of 1–35 was isolated previously from Zhuyeqing Liquor by author, structures of which were elucidated by comparison of spectral data (UV, MS, ^1^H NMR and ^13^C NMR) with the literature data (see Additional file 1: page S7, Reference). The purity of each reference standard was determined to be above 98%.

Bifendate drop pill (1.5 mg bifendate per pill) was provided by Beijing Union Pharmaceutical Plant.

Zhuyeqing Liquor (amber powder) was provided by Shanxi XinghuaCun Fen Jiu Group Co., Ltd. (Shanxi Province, China), The voucher specimen was deposited at Shenyang Pharmaceutical University (Shenyang, China) and registered under the number ZYQL 2011050101. Proper amount of Zhuyeqing liquor were evaporated in vacuum at 50°C to dryness. The dry residues was dissolved in sterilized distilled water before oral administration to the experimental animals. All doses given are the gram weight of the administered ZYQL powder in sterilized distilled water.

### HPLC analysis

ZYQL was analyzed using HPLC-PDA, proper amount of dry residues were dissolved with 70% ethanol to precipitate the polysaccharide in order to obtain better analytical results, then samples were filtrated through a 0.45 μm membrane filter. HPLC analysis was performed on a Waters 2695 Alliance HPLC system with Waters 2998 PDA detector.

Chromatographic analysis was performed on a SHIMADZU VP-ODS column (150 mm × 4.6 mm I.D. 5 μm). The mobile phase was methanol (A) and 0.1% Phosphoric acid (B). A gradient programmer was used according to the following profile: 0 min 5% A, 67 min 55% A, 75 min 60% A, 110 min 80% A, 120–125 min 98% A, 127–140 min 5% A. A constant flow rate of 1.0 ml/min was maintained through out the analysis. The column temperature was set to 25°C. Peaks were detected at 254 nm of PDA detection.

### UPLC-TOF-MS analysis

Waters UPLC-TOF-MS (Waters ACQUITY UPLC™ system tandem Waters LCT Premier XE TOF-MS, Software version: Mass Lynx V4.1) was used for the qualitative analysis. Chromatographic separation was carried on a ACQUITY UPLC BEH C_18_ (2.1 mm × 50 mm, 1.7 μm) at 30°C. Mobile phase was composed of acetonitrile (A) and 1% formic acid-water (B). The gradient program was as follows: 0–2.00 min, 30-95% A; 2.00-6.50 min, 95-95% A; 6.50-6.70 min, 95-30% A; 6.70-8.00 min, 30% A. The flow rate of mobile phase was set at 0.2 mL/min. The injection volume was 2 μL. For the MS analysis, nitrogen was used as desolvation gas at flow rates of 400 L/h for both ESI (+) and ESI (−). The cone gas flow rate was set at 10 L/h. The desolvation temperature was fixed at 200°C. The source temperature was set at 100°C. Capillary voltage was 2600 V for ESI (+) and 1800 V for ESI (−). The spectra were recorded in the range of m/z 100–1500 for full scan.

### Animals

Male Kunming strain mice (18 ± 2 g) were obtained from Experimental Animal Center of Shenyang Pharmaceutical University, China. The animals were housed for one week under controlled conditions before the experiments. These conditions were as follows: light (12 h light/dark cycle), temperature (24 ± 1°C), humidity (50 ± 5%), free access to food and water. The use of these animals was approved by the institute ethnics committee of Shenyang Pharmaceutical University. And the studies reported in this manuscript were carried out strictly in accordance with the guide for the care and use of laboratory animals (National Research Council of USA, 1996) and related ethical regulations of Shenyang Pharmaceutical university.

### Experimental design

After 1 week acclimatization, the animals were randomly divided into six equal groups as follows: normal control group, alcohol group, three groups for the high dose, medium dose and low dose of ZYQL and the positive control group for bifendate, each experimental group consisted of 12 mice.

For ZYQL treated group, the daily doses of ZYQL (high dose, medium dose and low dose) were 200, 100 and 50 mg/kg, respectively, other groups received an equal volume of vehicle as control. For positive control group, mice were received 150 mg/kg bifendate. By referring to related literatures about the dose of animal [10-12], all animals were administered orally with 50% alcohol 12 ml/kg at 4 h after the doses of ZYQL and bifendate, respectively everyday except mice in normal control group for fourteen consecutive days. Six hours after the last alcohol treatment, blood was first obtained from the animal through the orbital venous plexus, using capillary tubes, blood was allowed to clot and the serum was separated by centrifugation at 3000 rpm for 10 min at 4°C, and then stored at −80°C. Serum was used to analyze activity of liver enzymes ALT, AST and TBIL. Then, all the animals were sacrificed by cervical decapitation to obtain the liver for histopathological, immunohistochemical and biochemical assays.

### Biochemical assays

#### Measurement of liver function markers

Serum alanine aminotransferase (ALT), aspartate aminotransferase (AST) and TBIL (total bilirubin) were measured using Olympus kits (Olympus Corp., Tokyo, Japan) in an Olympus AU 600 Autoanalyzer. The observation absorbance of ALT and AST were read at 505 nm and the enzyme activity was calculated as U/L. The observation absorbance of TBIL was read at 600 nm and the content was calculated as μmol/L.

#### Measurement of malondialdehyde (MDA) formation in lipid peroxidation

The anti-lipid peroxidation was estimated by the method of Ohkawa (1979). Liver homogenate (10%, w/v) was prepared by homogenizing the liver tissue in 150 mM Tris–HCl buffered saline (pH 7.2) with a polytron homogenizer. The level of MDA in liver tissues was measured with a spectrophotometer (Hitachi U-2001) at 532 nm. The protein content was determined by the method of Lowry (1951). and the data are expressed as nmol MDA per milligram of protein of liver tissue (nmol/mg protein).

#### Measurement of hepatic GSH level

The measurement of GSH was conducted by modified protocol provided by GSH kit from Jiancheng Biological Engineering Institute (Nanjing, China). The observation absorbance of the reaction was read at 420 nm and the enzyme activity was calculated as mg/g protein.

#### Measurement of hepatic SOD activity

SOD activity was determined as described by Beauchamp (1971) by measuring its ability to inhibit the photochemical reduction of nitro blue tetrazolium (NBT) in absorbance at 550 nm. Following the commercial kit protocol provided by Jiancheng Biological Engineering Institute (Nanjing, China), Data was expressed as SOD units/mg protein as compared with the standard.

#### Histopathological observation

Mice liver specimens were fixed with 10% formaldehyde and processed routinely for embedding in paraffin, cut into 5 μm thick sections, stained with hematoxylin-eosin (H&E) for routine histopathological examination, and then examined under the light microscope.

#### Immunohistochemistry analysis

Formalin-fixed, paraffin-embedded sections (5 μM) were mounted on glass slides. Sections were deparaffinized, incubated in 3% H_2_O_2_ for 10 min to quench endogenous peroxidase activity. After blocking with normal goat serum for 20 min, the sections were stained with polyclonal rabbit anti-TNF-α antibody at 4°C overnight respectively, followed by incubation with horseradish peroxidase-conjugated goat anti-rabbit antibody at 37°C for 30 min. The antibody biding sites were visualized by incubation with DAB-H_2_O_2_ at room temperature for 10 min. Images were taken at original magnification of 200× (Olympus BX-50 Microscope, Japan and Leica DMIL, Leica Microsystems, Germany).

### Statistical analysis

Results were expressed as mean ± S.D. and all statistical comparisons were made by means of a one-way ANOVA test followed by Dunett’s t-test. *p* < 0.05 and <0.01 were considered statistically significant.

## Results

### HPLC profiles of Zhuyeqing liquor

**Figure 1:**
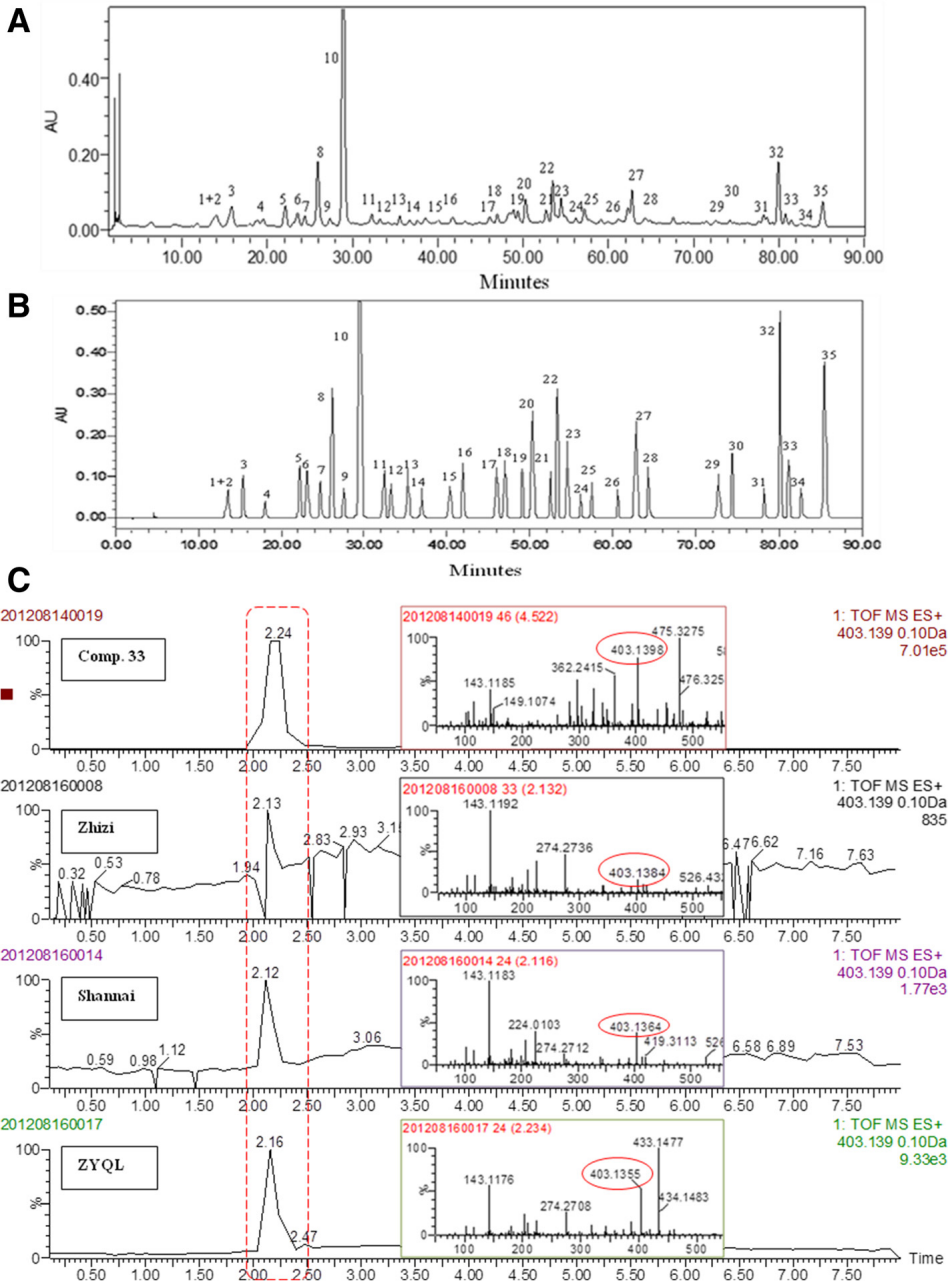
**Chromatograms of Zhuyeqing Liquor (ZYQL) and the standard reference. (A)** HPLC-PDA chromatogram of ZYQL monitored at 254 nm; **(B)** HPLC-PDA chromatogram of standard reference monitored at 254 nm. **(C)** Representative UPLC-TOF-MS positive ion mode chromatogram (From up to down: Compound 33, Zhizi, Shannai, Zhuyeqing Liquor).

The main compounds of ZYQL were analyzed with HPLC. The HPLC chromatogram and the representative UPLC-TOF-MS positive ion mode chromatogram showed in Figure 1 (A, B, C). By referring to standards, the chromatographic analysis showed the main components of ZYQL were 3-hydroxy-4,5(R)-dimethyl-2(5H)-furanone (**1**), (E)-2-hydroxy-3-methyl-penta- 3-enioc acid (**2**), protocatechuic aldehyde (**3**), picrocrocinic acid (**4**), isobiflorin (**5**), vanillic acid (**6**), biflorin (**7**), genipin 1-O-*β*-D-gentiobioside (**8**), 1-sinapoyl-*β*-D-glucopyranoside (**9**), geniposide (**10**), epijasmnoside A (**11**), kaempferol (**12**), rehmapicrogenin (**13**), ferulic acid (**14**), luteolin-8-C-*β*-glucopyranoside (**15**), isoorientin (**16**), narirutin (**17**), 10-O-acetylgeniposide (**18**), isorhamnetin-3-O-rutinoside (**19**), hesperidin (**20**), 6″-O-trans-*p*-coumaroyl genipin gentiobioside (**21**), 6″-O-cis-*p*-coumaroyl genipin gentiobioside (**22**), 2α-hydroxy oleanolic acid (**23**), 6′-O-(3-methoxyl caffeoyl)-epijasminoside B (**24**), 6′-O-sinapoylgeniposide (**25**), cis-*p*-coumaric acid ethyl ester (**26**), methyl 5-O-caffeoyl-3-O-sinapoylquinate (**27**), trans-*p*-coumaric acid ethyl ester (**28**), 3′,4′,5,6,7-pentamethoxyl flavone (**29**), 3,5-dihydroxy-3′,4′,7,8-tetramethoxyl flavone (**30**), 5-hydroxy-4′,6,7,8-tetramethoxyl flavone (**31**), 3′,4′,5,6,7,8-hexamethoxyl flavone (**32**), 3′,4′,3,5,6,8-hexamethoxyl flavone (**33**), kaempferide (**34**), oleanolic acid (**35**). (Structures of compound **1**–**35** see Additional file 1: Figure S1. Plant source and chromatographic analysis of these components were shown in Additional file 1: Table S1).

### General observation

All mice behaved excited except the normal group after administered orally with 50% alcohol 12 ml/kg, then showed unsteadily walking and accelerated breathing. Less activity was observed after 2–3 days. On the 14th day, mortality was observed in all groups except the normal group, and the group of ZYQL (200 mg/kg) exhibited the lowest death rate.

### Effect of ZYQL on organ coefficient

Liver, spleen and kidney coefficients were evaluated in mice. Compared to the control group, liver coefficients of alcohol group was significantly increased in mice (*p* < 0.05, Table 1); In contrast, there was no significant difference in spleen and kidney coefficient among all treated groups. As demonstrated in Table 1, the increase of liver coefficient caused by alcohol treatment in mice was reduced by ZYQL (200 mg/kg) and Bifendate (150 mg/kg), respectively (*p* < 0.01, *p* < 0.05, Table 1), and dose-effect correlation was observed in ZYQL groups regarding to the increase of liver coefficient by alcohol treatment. These results are presented in Table 1.

**Table 1:** Effect of ZYQL on organ coefficient in acute alcohol-induced liver injury in mice

**Groups**	**Dose(mg/kg)**	**Liver coefficient%**	**Kidney coefficient%**	**Spleen coefficient%**
Control	-	4.41 ± 0.53	1.46 ± 0.16	0.22 ± 0.07
Alcohol	-	5.14 ± 0.27^a^	1.40 ± 0.17	0.29 ± 0.11
ZYQL-High	200	4.29 ± 0.83^c^	1.45 ± 0.15	0.23 ± 0.05
ZYQL-Medium	100	4.56 ± 1.02	1.46 ± 0.13	0.24 ± 0.07
ZYQL-Low	50	4.58 ± 0.82	1.44 ± 0.09	0.27 ± 0.03
Bifendate	150	4.40 ± 0.35^b^	1.40 ± 0.06	0.28 ± 0.04

Values represent the mean ± S.D. The one-way ANOVA was performed on the raw data.

^*a*^Significant difference at *p* < 0.05 levels compared with the control group.

^*b*^Significant difference at *p* < 0.05 levels compared with alcohol treated group.

^*c*^Significant difference at *p* < 0.01 levels compared with alcohol treated group.

### Effect of ZYQL on ALT, AST and TBIL levels

The serum activities of ALT, AST and TBIL were used as biochemical markers for the early acute hepatic damage. In Table 2, serum ALT, AST and TBIL were increased by 2.04-, 2.20- and 3.20- fold, respectively over those in normal mice after administration of alcohol on the 14th day. Pretreatment with 50, 100 and 200 mg/kg of ZYQL significantly reduced the elevation of ALT, AST and TBIL (*p* < 0.01, *p* < 0.05) and in a dose-dependent manner. Bifendate pretreatment also offered significant (*p* < 0.01) protection against acute alcohol-intoxicated mice by attenuating ALT, AST and TBIL elevation (Table 2).

**Table 2:** Effect of ZYQL on ALT, AST and TBIL levels in acute alcohol-induced liver injury in mice

**Groups**	**Dose(mg/kg)**	**ALT**^**d**^	**AST**^**d**^	**TBIL**^**e**^
Control	-	32.34 ± 8.81	44.81 ± 19.48	6.76 ± 0.73
Alcohol	-	66.03 ± 15.02^a^	98.73 ± 18.29^a^	21.62 ± 2.03^a^
ZYQL-High	200	33.30 ± 9.40^c^	47.47 ± 9.09^c^	6.33 ± 1.95^c^
ZYQL-Medium	100	34.85 ± 8.12^c^	50.55 ± 11.07^c^	9.84 ± 1.45^c^
ZYQL-Low	50	46.16 ± 6.92^b^	65.16 ± 10.33^b^	12.59 ± 1.40^b^
Bifendate	150	24.74 ± 5.30^c^	47.27 ± 8.95^c^	6.96 ± 1.41^c^

Values represent the mean ± S.D. The one-way ANOVA was performed on the raw data.

^*a*^Significant difference at *p* < 0.01 levels compared with the control group.

^*b*^Significant difference at *p* < 0.05 levels compared with alcohol treated group.

^*c*^Significant difference at *p* < 0.01 levels compared with alcohol treated group.

^*d*^IU/L.

^*e*^μmol/L.

### Effect of ZYQL on MDA, GSH and SOD levels

MDA is an end-product of the breakdown of polyunsaturated fatty acids and related esters, and its formation is an index of lipid peroxidation in many organ homogenate [13]. As shown in Table 3, the levels of hepatic MDA significantly increased in alcohol group (*p* < 0.01), alcohol-induced elevation of tissue MDA concentration was lowered significantly (*p* < 0.01, *p* < 0.05) by pretreated with ZYQL (50, 100 and 200 mg/kg) with a dose-dependent manner (7.58 ± 1.79, 5.63 ± 1.11 and 4.41 ± 1.23). Hepatic GSH and SOD levels were decreased after alcohol treatment (*p* < 0.01, Table 3). Pretreatment with ZYQL (100 and 200 mg/kg) exhibited protection against alcohol-induced hepatic GSH and SOD depletion. The low dose of ZYQL (50 mg/kg) showed obvious protective effect against alcohol-induced decrease of SOD levels (*p* < 0.05), while no obvious effect on GSH.

**Table 3:** Effect of ZYQL on MDA, SOD and GSH levels in acute alcohol-induced liver injury in mice

**Groups**	**Dose(mg/kg)**	**MDA**^**d**^	**SOD**^**e**^	**GSH**^**f**^
Control	-	3.72 ± 1.09	301.68 ± 24.93	4.34 ± 1.33
Alcohol	-	9.04 ± 1.48^a^	188.30 ± 22.89^a^	0.85 ± 0.30^a^
ZYQL-High	200	4.41 ± 1.23^c^	290.56 ± 15.13^c^	3.73 ± 1.53^c^
ZYQL-Medium	100	5.63 ± 1.1 ^c^	255.49 ± 12.16^c^	2.02 ± 1.10^b^
ZYQL-Low	50	7.58 ± 1.79^b^	215.86 ± 6.41^b^	1.64 ± 0.70
Bifendate	150	4.60 ± 0.97^c^	267.56 ± 26.13^c^	2.58 ± 0.91^c^

Values represent the mean ± S.D. The one-way ANOVA was performed on the raw data.

^*a*^Significant difference at *p* < 0.01 levels compared with the control group.

^*b*^Significant difference at *p* < 0.05 levels compared with alcohol treated group.

^*c*^Significant difference at *p* < 0.01 levels compared with alcohol treated group.

^*d*^nmol/mgprot.

^*e*^U/mgprot.

^*f*^mg/gprot.

### Histopathological studies

Observed by naked eyes, the livers of vehicle control group were deep red, moist, glossy and resilient. In alcohol group, the livers lost luster and yellow necrosis foci were often found on the surface. Liver injury of ZYQL pretreated mice was attenuated dramatically in a dose-dependent manner (Additional file 1: Figure S2).

**Figure 2:**
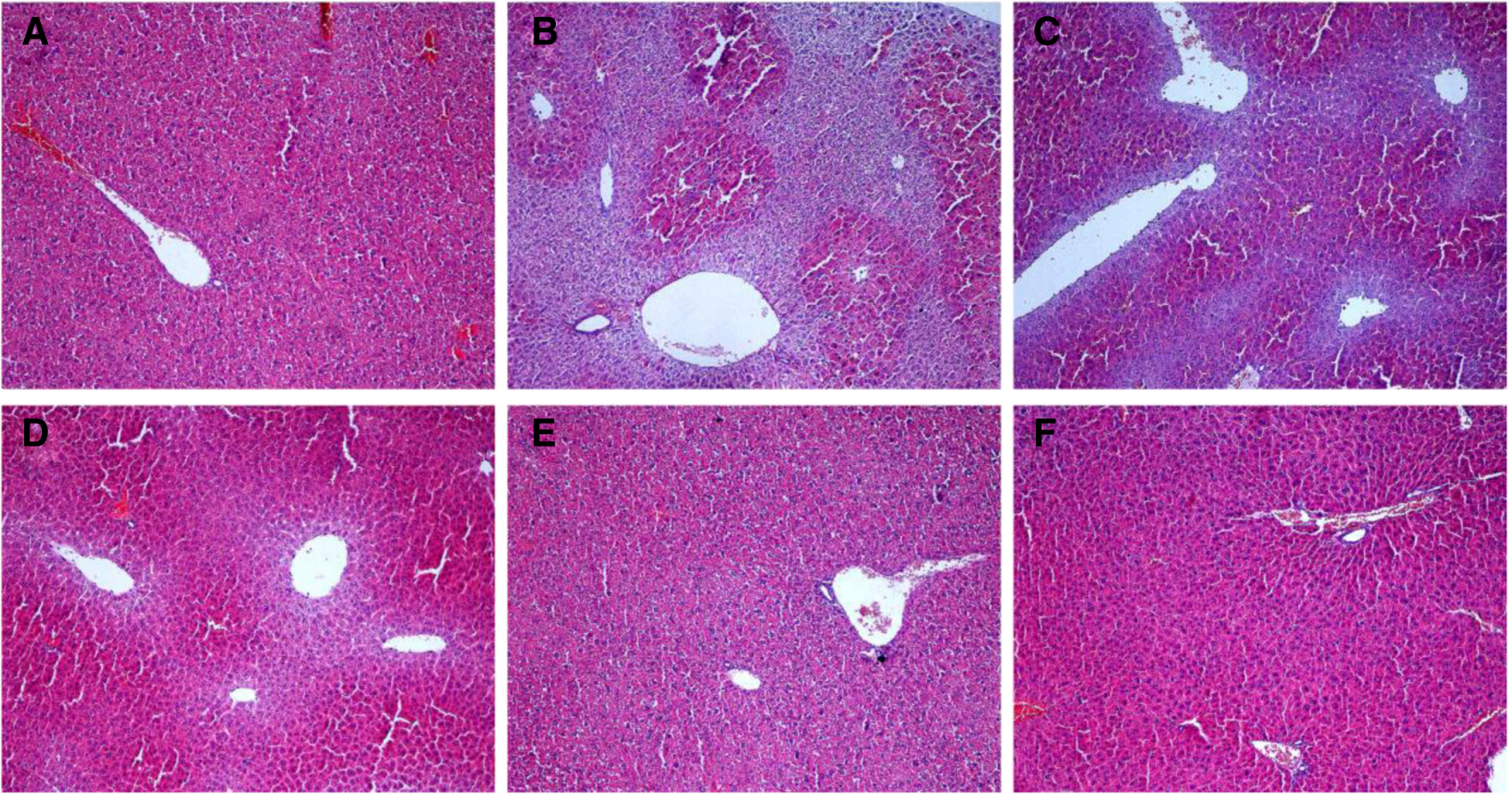
**Hepatoprotective effect of Zhuyeqing Liquor on Alcohol-induced hepatotoxicity in mice.** Liver sections were stained with haematoxylin and eosin (×100). **(A)** Normal control group; **(B)** Alcohol-induced group; **(C)**, **(D)** and **(E)** are Alcohol group treated with 50, 100 and 200 mg/kg of ZYQL, respectively; **(F)** is Alcohol group treated with 150 mg/kg of Bifendate.

The histological features under light microscope, as shown in Figure 2A, indicated a normal liver lobular architecture and cell structure of the livers in the control animals, which showed that the liver lobular structures were clear and regular, and single layer of hepatocytes arranged around the central vein in a radical pattern and there were abundant basophilic granular cytoplasms in the hepatocytes. In alcohol treated group, normal liver lobular structures were damaged and collapsed. The hepatocytes showed vacuolization, sinusoidal dilation and congestion. Infiltration of inflammatory cells and loss of cell boundaries were also observed (Figure 2B). Histopathologcial changes induced by alcohol were remarkably improved by ZYQL, the high dose of ZYQL alleviated the lipid change of hepatocytes (Figure 2C), showing obvious difference from the model group and similar to bifendate group (Figure 2F).

### Immunohistochemistry analysis

**Figure 3:**
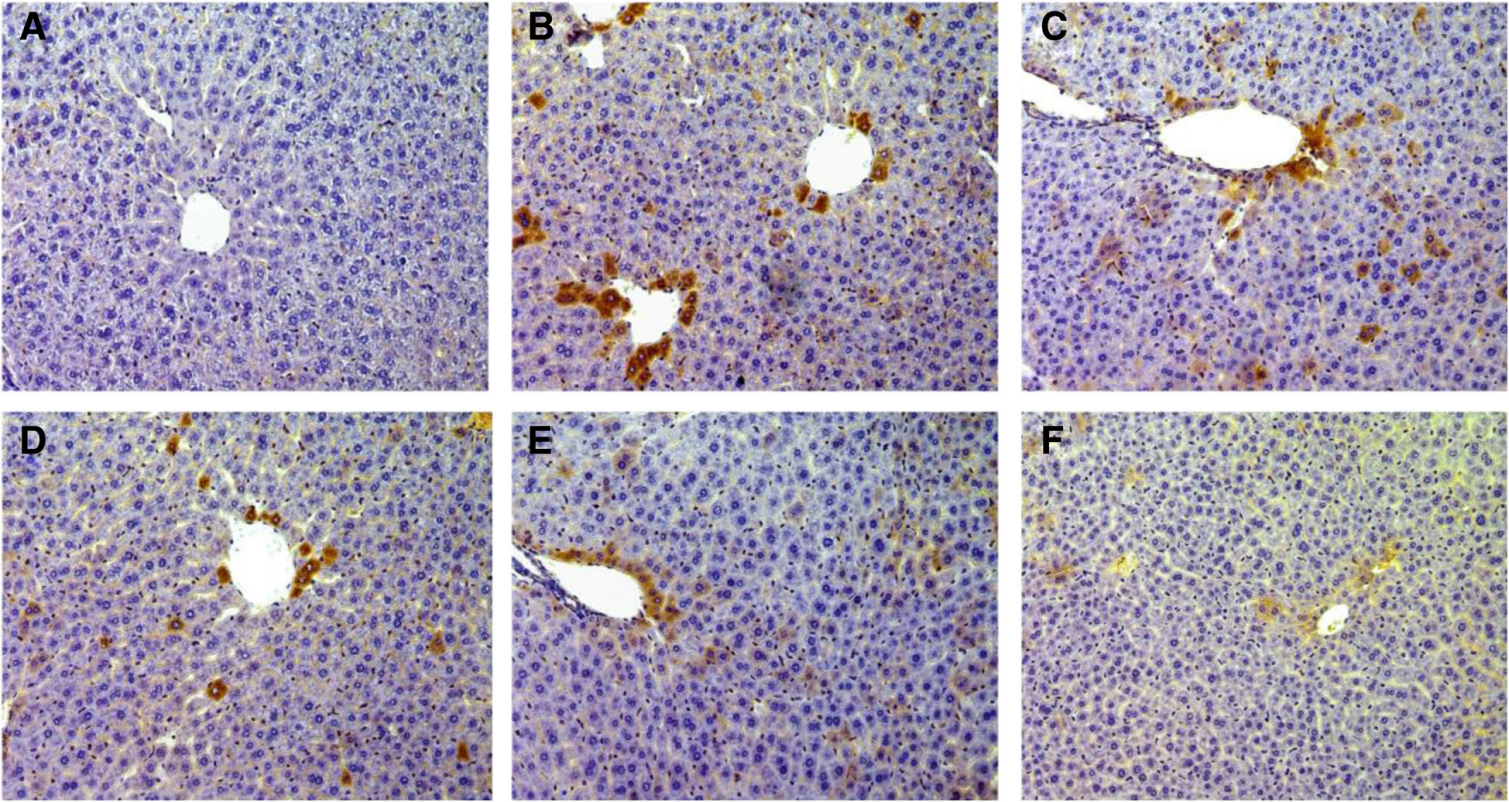
**Representative photographs of immunohistochemical localization of TNF-**α **in mice liver with Alcohol hepatotoxicity (×200). (A)** Normal control group; **(B)** Alcohol-induced group; **(C)**, **(D)** and **(E)** are Alcohol group treated with 50, 100 and 200 mg/kg of ZYQL, respectively; **(F)** is Alcohol group treated with 150 mg/kg of Bifendate.

Varying degrees of TNF-α expression were seen in the diseased liver tissues. The normal control group exhibited minimal TNF-α expression (Figure 3A), whereas all experimental groups showed increased expression of TNF-α, especially in the alcohol-intoxicated group (Figure 3B). After pretreatment with ZYQL (50, 100 and 200 mg/kg), TNF-α expression shows decrease but it is still higher than normal group (Figures 3C, D, E). While similar to previous study, pretreatment with Bifendate (150 mg/kg), an agent being applied clinically to treat liver disease, abolishes the positive signals of TNF-α (Figure 3F).

## Discussion

Alcohol-induced liver injury, one of the most common causes of liver diseases worldwide, has been paid more attention and which presents initially as acute inflammation then progresses to fatty liver, alcoholic hepatitis and ultimately to fibrosis and cirrhosis [14]. Binge alcohol drinking is a more potent health hazard caused due to bulk intoxication of alcohol at a single go. However, presently there is no universally accepted therapy available to halt or reverse this process in humans [15]. Thus, more attention has been paid to the research and development of effective therapy for ALD and agents for protecting alcohol-induced liver injury. At present, it has been recognized that oxidative stress and generation of free radicals play a critical role in the development of ALD. Therefore, some natural products with antioxidant activity have attracted great attention as potential functional ingredients to protect alcohol-induced liver injury [16,17].

In China, ZYQL as a functional health liquor has been exist for more than a millennium, and the long time history use of ZYQL has been proved that drinking of ZYQL could improve body immunity. Moreover, recent reports also suggest that ZYQL has the functions of anti-oxidation, anti-fatigue and body immunoenhancement [4]. However, whether ZYQL could alleviate the damage comes from liquor drinking is not clear. Thus, in the present study, we investigated the hepatoprotective effects of ZYQL on acute alcohol-induced liver injury in mice. In this present study, our results confirmed the involvement of oxidative stress in acute alcohol-induced liver injury and showed significant protective effect of ZYQL, as evidenced by decreasing the level of MDA and inhibiting the decrease of SOD and GSH activity. The attenuation of acute alcohol-induced oxidative stress by ZYQL was probably due to its ability to restore the balance between generation and clearance of ROS. Reduction in the levels of AST, ALT and TBIL towards the normal value was an indication of the stabilisation of plasmamembrane and the repair of hepatic tissue. Moreover, Histological examination of the liver sections revealed lipid change of hepatocytes treated with alcohol, while in the sections obtained from the mice treated with ZYQL and with alcohol, the lipid change of hepatocytes was alleviated. Thus, these results suggested that the inhibition of liver function markers elevation and liver damage may participate in the protective effect of ZYQL against alcohol-induced hepatotoxicity.

By using HPLC analysis, we found that the main components of ZYQL were iridoids, flavonoids, terpenoids and phenolic acids. In the study, pretreatment with ZYQL exhibited a significant decrease in the elevated AST, ALT, and TBIL (*p* < 0.01, *p* < 0.05) levels in a dose-dependent manner, this indicated that ZYQL pretreatment offered significant protection against acute alcohol-intoxicated mice, and can also relieve the damage of alcohol on the cell membrane and mitochondria membrane. This effect is in agreement with the commonly accepted view that serum levels of transaminases return to normal with the healing of hepatic parenchyma and regeneration of hepatocytes [18].

Production of ROS during alcohol-induced liver injury is the major causative factor for the liver damage [19]. To address this point, experiments were performed to measure lipid peroxidation (MDA), SOD and GSH content in liver tissues of the control and the experiment group. Lipid peroxidation (MDA) were significantly increased by 2.4-fold in alcohol-treated tissues as compared to control. Alcohol-treated liver tissue exhibited 1.6-fold less SOD and 5.1-fold less GSH compared to untreated ones (Table 3). Moreover, pretreatment with ZYQL enhanced the activities of antioxidant enzyme (SOD), increased the antioxidant content (GSH), and diminished the amount of lipid peroxide (MDA) against the alcohol-induced hepatotoxicity in these animals, suggesting that the activity of antioxidant and decreasing the formation of lipid peroxidation may plays a role in the mechanism of its hepatoprotective effects.

Tumor necrosis factor (TNF-α)-α is a central proinflammatory cytokine, and it has been suggested that it is important in the development of alcohol induced liver injury [20]. Because TNF-α is produced predominantly by the monocyte macrophage lineage and the major population of this lineage in the liver is Kupffer cells [21], increased production of TNF-α by activated Kupffer cells may be responsible for alcoholic hepatitis. Recent studies have shown that inhibition of TNF-α could decrease in the amount of hepatic fatty storage in alcohol-treated mice [22]. In the present study, the effects of TNF-α in the damaged liver was evaluated by immunohistochemistry. Compared to the control group, treated with alcohol upregulated the expression of TNF-α, while pretreatment with ZYQL downregulated the expression of TNF-α compared to the alcohol-intoxicated group.

Recent research findings about the hepatoprotective and antioxidant effect of components in twelve single herbs of ZYQL have been reported. Oleanolic acid in *Eugenia caryophyllata* Thunb. (Dingxiang), an agent being applied clinically to treat liver disease, significantly reduced the serum level of AST and ALT increased by CCl_4_ intoxication [23]. Geniposide in *Gardenia jasminoides* Ellis (Zhizi) could significantly reduce the level of MDA increased by alcohol. Sudnikovich et al. reported that flavonoids inhibit peroxidation by acting as chain breaking peroxyl-radical scavengers [24]. Kaempferol, luteolin-8-C-β-glucopyranoside, isoorientin, narirutin, isorhamnetin 3-O-rutinoside, hesperidin, 3′,4′,5,6,7-pentamethoxyl flavone, 3,5-dihydroxy-3′,4′,7,8- tetramethoxyl flavone, 5-hydroxy-4′,6,7,8-tetramethoxyl flavone, 3′,4′,5,6,7,8-hexamethoxyl flavone, 3′,4′,3,5,6,8-hexamethoxyl flavone, kaempferide were flavonoids found in *Angelica sinensis* (Oliv.) Diels (Danggui), *Kaempferia galanga* L. (Shannai), *Citrus reticulata* Blanco (Chenpi), *Chrysanthemum morifolium* Ramat. (Juhua) and *Aucklandia lappa* Decne. (Muxiang) exhibited antioxidant activity and increased the metabolism of alcohol according to the literature reported [8,9,25,26]. Phenolic compounds are commonly found in both edible and other traditional medicinal plants, and they have been reported to have multiple biological activities, including free radical scavenging activity [27-29]. Protocatechuic aldehyde, vanillic acid, ferulic acid, cis-*p*-coumaric acid ethyl ester and trans-*p*-coumaric acid ethyl ester were phenolic compounds found in *Lophatherum gracile* Brongn. (Zhuye), *Amomum villosum* Lour. (Sharen), *Santalum album* L. (Tanxiang) and *Lysimachia foenum-graecum* Hance (Linglingxiang) also exhibited antioxidant and radical scavenging activities [29]. The HPLC, UPLC-TOF-MS analysis and the hepatoprotective effect of ZYQL in our study suggested that the activity of ZYQL may be related to compounds including iridoids, flavonoids, terpenoids and phenolic acid from the twelve single herbs. Therefore, we proposed that iridoids, flavonoids, terpenoids and phenolic acids may be used for the quality control of ZYQL. Further research elucidating the action mechanism of these effects using purified ingredients and single herbs will give an insight into the usefulness of this prescription in the protective effect on the acute alcohol-induced liver injury.

## Conclusion

Taken together, the present study suggests that ZYQL has a potent hepatoprotective activity in alcohol-induced liver injury in mice, especially the dose of 200 mg/kg. The enhanced levels of antioxidant enzymes, reduced amount of lipid peroxides, ameliorate hepatic function and the suppression of TNF-α production in liver are suggested to be the major mechanisms of ZYQL in preventing the development of liver damage induced by alcohol. Phytochemical analysis also showed that the mainly active chemical constitutes in ZYQL included iridoids, flavonoids, terpenoids and phenolic acids. Our investigation provided convincing data supporting the potential function of health care and the quality control of ZYQL.

## Competing interests

The authors declare that they have no competing interests.

## Authors’ contributions

The author, HYG, HYW, SMC carried out the whole experiment. XWD, YH, LFW prepared the experimental drug. HYG and JHW participated in the design of the study, performed the statistical analysis and drafted the manuscript. All authors read and approved the final manuscript.

## Supplementary Material

Additional file 1 Reference, Figure S1, Table S1 and Figure S2. Click here for file
