# Supplementary material for: Protective effect of Zhuyeqing liquor, a Chinese traditional health liquor, on acute alcohol-induced liver injury in mice
Source: J Inflamm (Lond). 2013 Oct 3;10:30. doi: 10.1186/1476-9255-10-30 (PMC3851207; doi:10.1186/1476-9255-10-30)
Supplement: Additional file 1 — Reference, Figure S1, Table S1 and Figure S2. [file 1476-9255-10-30-S1.docx]

**Protection of the Zhuyeqing Liquor, a Chinese traditional health Liquor, on acute alcohol-induced liver injury in mice**

Hong-ying Gao^1^, Jian Huang ^1^, Hang-yu Wang ^2^, Xiao-wei Du ^3^, Suo-ming Cheng^2^, Ying Han^3^, Li-fei Wang^3^, Guo-yu Li^2, *^, Jin-hui Wang^1, 2,^ ^[[1]](#footnote-1)^*

1) School of Traditional Chinese Materia Medica 49^#^, Shenyang Pharmaceutical University, Wenhua Road 103, Shenyang 110016, P. R. China (phone: +86-24-23986479; fax: +86-24-23986479; e-mail: [wjh.1972@aliyun.com](mailto:wjh.1972@aliyun.com.cn))

2) School of Pharmacy, Shihezi University, Shihezi 832002, P. R. China

3) Shanxi Xinghuacun Fen Jiu Group Co., Ltd., 450000 Shanxi, P. R. China

| **No** | **Contents** | **Page** |
| --- | --- | --- |
| 1 | Plant source and chromatographic analysis of Compounds **1-35** | S3 |
| 2 | Structures of Compounds **1**-**35** | S4 |
| 3 | The appearance morphous of mice liver | S6 |
| 4 | Reference of compounds **1-35** | S7 |

**Table S1** Plant source and chromatographic analysis of compounds **1-35**

| No. | T_R_(min)^a^ | Compounds name | Crude Drug |
| --- | --- | --- | --- |
| 1 | 13.78 | 3-hydroxy-4,5(*R*)-dimethyl-2(5*H*)-furanone | Zhuye, Paicao |
| 2 | 13.78 | (*E*)-2-hydroxy-3-methyl-penta-3-enioc acid | Zhuye, Paicao |
| 3 | 15.34 | Protocatechuic aldehyde | Sharen, Linglingxiang |
| 4 | 18.32 | Picrocrocinic acid | Zhizi, Linglingxiang |
| 5 | 22.01 | Isobiflorin | Dingxiang |
| 6 | 23.11 | Vanillic acid | Sharen, Dingxiang |
| 7 | 24.50 | Biflorin | Dingxiang |
| 8 | 25.92 | Genipin 1-O-*β*-D-gentiobioside | Zhizi |
| 9 | 27.47 | 1-sinapoyl-*β*-D-glucopyranoside | Dingxiang |
| 10 | 29.12 | Geniposide | Zhizi |
| 11 | 32.23 | Epijasmnoside A | Zhizi, Dingxiang |
| 12 | 33.09 | Kaempferol | Shannai |
| 13 | 35.29 | Rehmapicrogenin | Muxiang |
| 14 | 36.93 | Ferulic acid | Dingxiang, Danggui |
| 15 | 40.16 | Luteolin 8-C-*β*-glucopyranoside | Zhuye, Muxiang, Dingxiang |
| 16 | 41.75 | Isoorientin | Juhua, Muxiang, Chenpi |
| 17 | 46.05 | Narirutin | Juhua, Muxiang |
| 18 | 47.08 | 10-O-acetylgeniposide | Zhizi |
| 19 | 49.24 | Isorhamnetin 3-O-rutinoside | Chenpi |
| 20 | 50.09 | Hesperidin | Chenpi |
| 21 | 52.75 | 6''-O-trans-*p*-coumaroyl genipin gentiobioside | Zhizi |
| 22 | 53.42 | 6''-O-cis-*p*-coumaroyl genipin gentiobioside | Zhizi |
| 23 | 54.37 | 2α-hydroxy oleanolic acid | Tanxiang |
| 24 | 56.01 | 6′-O-(3-methoxyl caffeoyl)-epijasminoside B | Zhizi |
| 25 | 57.17 | 6′-O-sinapoylgeniposide, | Zhizi |
| 26 | 61.61 | Cis-*p*-coumaric acid ethyl ester | Zhuye, Juhua,Dingxiang |
| 27 | 62.89 | Methyl 5-O-caffeoyl-3-O-sinapoylquinate | Muxiang |
| 28 | 64.49 | Trans-*p*-coumaric acid ethyl ester | Zhuye, Juhua |
| 29 | 72.65 | 3′,4′,5,6,7-pentamethoxyl flavone | Dingxiang, Chenpi |
| 30 | 74.36 | 3,5-dihydroxy-3′,4′,7,8-tetramethoxyl flavone | Zhizi, Chenpi |
| 31 | 78.59 | 5- hydroxy-4′,6,7,8- tetramethoxyl flavone | Chenpi |
| 32 | 80.09 | 3′,4′,5,6,7,8-hexamethoxyl flavone | Zhizi, Danggui, Chenpi |
| 33 | 81.14 | 3′,4′,3,5,6,8-hexamethoxyl flavone | Zhizi, Shannai |
| 34 | 82.94 | Kaempferide | Shannai |
| 35 | 85.53 | Oleanolic acid | Muxiang, Dingxiang |

^a^ Peaks were detected at 254 nm of PDA detection.

**Figure S1**

**Fig. S1** The structure of compound **1-35**

**Figure S2**

Observed by naked eyes, the livers of vehicle control group were deep red, moist, glossy and resilient. In alcohol group, the livers lost luster and yellow necrosis foci were often found on the surface. Liver injury of ZYQL pretreated mice was attenuated dramatically in a dose-dependent manner (Fig. S2).

**Fig. S2** The appearance morphous of experimental mice liver. (A) Normal control group; (B) Alcohol-induced group; (C), (D) and (E) are Alcohol group treated with 50, 100 and 200 mg/kg of ZYQL, respectively; (F) is Alcohol group treated with 150 mg/kg of Bifendate.

**Reference**

Anh, N.T. H., Sung, T.V., Franke, K., Wessjohann, L. A., 2003. Phytochemical studies of Rehmannia glutinosa rhizomes. Pharmazie 58, 593-595.

Ballester, A.R., Lafuente, M. T., de Vos, R.C.H., Bovy, A.G., González-Candelas, L., 2013. Citrus phenylpropanoids and defence against pathogens. Part I: Metabolic profiling in elicited fruits. Food Chemistry 136, 178-185.

Chen, Q.C., Youn, U.J., Min, B.S., Bae, K.H., 2008. Pyronane Monoterpenoids from the Fruit of Gardenia jasminoides. Journal of Natrual Product 71, 995-999.

Cornelius, M.T. F., de Carvalho, M.G., da Silva, T.M. S., Alves, C.C. F., Siston, A.P. N., Alves, K.Z., Sant'Anna, C.M. R., Neto, M.B., Eberlin, M.N., Braz-Filho, R., 2010. Other chemical constituents isolated from Solanum crinitum Lam. (Solanaceae). Journal of the Brazilian Chemical Society 21, 2211-2219.

Dinda, B., Roy Chowdhury, D., Mohanta, B.C., 2009. Naturally Occurring Iridoids, Secoiridoids and Their Bioactivity. An Updated Review, Part 3. Chemical & Pharmaceutical Bulletin 57, 765-796.

Dinda, B., Debnath S., Banik, R., 2011. Naturally Occurring Iridoids and Secoiridoids. An Updated Review, Part 4. Chemical & Pharmaceutical Bulletin 59, 803-833.

[Esteban](http://www.sciencedirect.com/science/article/pii/S0031942200813254), M.D., [González Collado](http://www.sciencedirect.com/science/article/pii/S0031942200813254), L., [Macías](http://www.sciencedirect.com/science/article/pii/S0031942200813254), F.A., [Massanet](http://www.sciencedirect.com/science/article/pii/S0031942200813254), G.M., [Rodríguez Luis](http://www.sciencedirect.com/science/article/pii/S0031942200813254), F., 1986. Flavonoids from Artemisia lanata, [Phytochemistry](http://www.sciencedirect.com/science/journal/00319422) [25,](http://www.sciencedirect.com/science/journal/00319422/25/6) 1502-1504.

Fu, Z.H., Zhang, Y.M., Tan, N.H., Chu, H.B., Ji, C.J., 2008. Chemical constituents of Keteleeria evelyniana. Natural Product Research and Development 20, 257-261.

Hamdan, D., El-Readi, M.Z., Tahrani, A., Herrmann, F., Kaufmann, D., Farrag, N., El-Shazly, A., Wink, M., 2011. Chemical composition and biological activity of Citrus jambhiri Lush. Food Chemistry 127, 394-403.

Huang, Y., Chang, R.J., Jin, H.Z., Zhang, W.D., 2012. Phenolic constituents from tsoongiodendron odorum chun. Tianran Chanwu Yanjiu Yu Kaifa , 24, 176-178.

Ke, Y., Jiang, Y., Luo, S.Q., 1999. Chemical constituents from Clinopodium chinense (Benth.) O. Ktze., Chinese traditional and herbal drugs 30, 8-10.

Kim, H.J., Kim, E.J., Seo, S.H., Shin, C.G., Jin, C., Lee, Y.S., 2006. Vanillic Acid Glycoside and Quinic Acid Derivatives from Gardeniae Fructus, Journal of Natural Products 69, 600-603.

Kumarasamy, Y., Byres, M., Cox, P.J., Delazar, A., Jaspars, M., Nahar, L., Shoeb, M., Sarker, S.D., 2004. Isolation, structure elutiondition, and biological activity of flavone 6-C-glycosides from Alliaria petiolata. Chemistry of Natural Compounds 40, 122-128.

Lin, L.B., Fu, X.W., AI, C.H., Shen, J., Wei, K., Li, W., 2006. Studies on chemical constituents in leaves of Mallotus furetianus. China Journal of Chinese Materia Medica 31, 477-479.

Liu, X.M., Jiang, Y., Sun, Y.Q., Xu, X.W., Tu, P.F., 2011. Chemical constituent study of Herba Cistanches. Chinese Journal of Pharmaceuticals 46, 1053-1058.

Ma, Z.T., Yang, X.W., Zhong, G.Y., 2009. A new flavonoid glucoside from Huanglian jiedutang decoction. China Journal of Chinese Materia Medica 34, 1097-1100.

Machida, K., Osawa, K., 1989. On the Flavonoid constituents from the Peels of Citrus hassaku HORT. ex TANAKA. Chemical & Pharmaceutical Bulletin 37, 1092-1094.

Machida, K., Onodera, R., Furuta, K., Kikuchi, M., 1998. Studies of the Constituents of Gardenia Species. I, Monoterpenoids from Gardeniae Fructus. Chemical & Pharmaceutical Bulletin 46. 1295-1300.

Miyake, Y., Mochizuki, M., Okada, M., Hiramitsu, M., Morimitsu, Y., Osawa, T., 2007. Isolation of Antioxidative Phenolic Glucosides from Lemon Juice and Their Suppressive Effect on the Expression of Blood Adhesion Molecules. Bioscience, Biotechnology, and Biochemistry 71, 1911-1919.

Okamurα, N., Hine, N., Tateyamα, Y., Nakazawα, M., Fujiokα, T., Mihashi, K., Yagi, A., 1998. Five chromones from Aloe Vera leaves. Phytochemistry 49, 219-223.

Rayyan, S., Fossen, T., Solheim Nateland, H., Andersen, Ø. M., 2005. Isolation and identification of flavonoids, including flavone rotamers, from the herbal drug ‘crataegi folium cum flore’ (hawthorn). Phytochemical Analysis 16, 334-341.

Sadtler Research Laboratories. 1989. Sadtler Standard Carbon-^13^NMR Spectra [Z]. Pennsylvania (USA): Sadtler Research Laboratories, 6246C.

Wang, G., Liu, J.S., Li, H.Y., Zhang, L., Liu, J.K., 2011. Chemical constituents of loropetalum chinense. Tianran Chanwu Yanjiu Yu Kaifa 23, 267-269.

Wang, H.S., Wang, Y.H., Shi, Y.N., Li, X.Y., Long, C.L., 2009. Chemical constituents in roots of Osbeckia opipara. China Journal of Chinese Materia Medica 34, 414-418.

Wang, Q.J., Wang, Y.S., He, L., Lou, Z.P., Zang, S., 2010. Study on chemical constituents from Ipomoea Pescaprae (L.) Sweet. Chinese Journal of Marine Drugs 29 41-44.

Wang, S.M., Hu, Q., Shen, J., 2012. Study on active constituents of Danxiang Guanxin Injection by GC/MS and UPLC/Q-TOF-MS. Zhongchengyao 34, 78-84.

Wu, L.J., Xiang, T., Hou. B.L., Liang, W., Yin, S., Zhou, X.C., 1998. Chemical Constituents from Fruits of Ligustrum lucidum. Acta Botanica Sinica 40, 83-87.

Xu, M.L., Moon, D.C., Lee, C.S., Woo, M.H., Lee, E.S., 2006. Cytotoxicity and DNA topoisomerase inhibitory activity of constituents isolated from the fruits of Evodia officinalis, Archives of Pharmacal Research 29, 541-547.

Yoo, S.W., Kim, J.S., Kang, S.S., Son, K.H., Chang, H.W., Kim, H.P., Bae, K.H., Lee, C.O., 2002. Constituents of the Fruits and Leaves of *Euodia daniellii*. Archibes of Pharmacal Research (Arch Pharm Res) 25, 824-830.

Yu, Y., Xie, Z.L., Gao, H., Ma, W.W., Dai, Y., Wang, Y., Zhong Y., Yao, X.S., 2009. Bioactive Iridoid Glucosides from the Fruit of Gardenia jasminoides. Journal of Natural Products 72, 1459-1464.

Zhang, Y.W., Chen, Y.W., 1997. Isobiflorin, A chromone c-glucoside from cloves (Eugenia Caryophyllata). Phytochemistry 45, 401-403.

1. * Corresponding to: Jin-Hui Wang, School of Traditional Chinese Materia Medica 49^#^, Shenyang Pharmaceutical University, Wenhua Road 103, Shenyang 110016, P. R. China. Tel.: +86-24-23986479; fax: +86-24-23986479.

   E-mail address: [wjh.1972@aliyun.com](mailto:wjh.1972@aliyun.com) (J.-H. Wang). [↑](#footnote-ref-1)
